# U-Shaped Association between Waist-to-Hip Ratio and All-Cause Mortality in Stage 3–5 Chronic Kidney Disease Patients with Body Mass Index Paradox

**DOI:** 10.3390/jpm11121355

**Published:** 2021-12-13

**Authors:** Feng-Ching Shen, Yi-Wen Chiu, Mei-Chuan Kuo, Ming-Yen Lin, Jia-Jung Lee, Shang-Jyh Hwang, Jer-Ming Chang, Chi-Chih Hung, Hung-Chun Chen

**Affiliations:** 1 Division of Nephrology, Department of Internal Medicine, Kaohsiung Medical University Hospital, Kaohsiung Medical University, Kaohsiung 80708, Taiwan; sam0927035368@gmail.com (F.-C.S.); chiuyiwen@kmu.edu.tw (Y.-W.C.); mechku@kmu.edu.tw (M.-C.K.); mingyenlin3@gmail.com (M.-Y.L.); jjlee@kmu.edu.tw (J.-J.L.); sjhwang@kmu.edu.tw (S.-J.H.); jemich@kmu.edu.tw (J.-M.C.); chenhc@kmu.edu.tw (H.-C.C.); 2 Faculty of Renal Care, College of Medicine, Kaohsiung Medical University, Kaohsiung 80708, Taiwan; 3 Regenerative Medicine and Cell Therapy Research Center, Kaohsiung Medical University, Kaohsiung 80708, Taiwan

**Keywords:** obesity paradox, body mass index, central obesity, advanced chronic kidney disease, waist-to-hip ratio, waist-to-height ratio, conicity index, all-cause mortality

## Abstract

The obesity paradox, referring to the association of high body mass index (BMI) with low all-cause mortality risk, is found in patients with chronic kidney disease (CKD). Central obesity is associated with metabolic syndrome and may have better prognostic value than BMI for all-cause mortality. Whether central obesity is associated with all-cause mortality in cases of obesity paradox in CKD patients remains unknown. We included 3262 patients with stage 3–5 CKD, grouped into five quintiles (Q1–5) by waist-to-hip ratio (WHR). Low WHR and BMI were associated with malnutrition and inflammation. In Cox regression, high BMI was not associated with all-cause mortality, but BMI < 22.5 kg/m^2^ increased the mortality risk. A U-shaped association between central obesity and all-cause mortality was found: WHR Q1, Q4, and Q5 had higher risk for all-cause mortality. The hazard ratio (95% confidence interval) of WHR Q5 and Q1 for all-cause mortality was 1.39 (1.03–1.87) and 1.53 (1.13–2.05) in male and 1.42 (1.02–1.99) and 1.28 (0.88–1.85) in female, respectively. Waist-to-height ratio and conicity index showed similar results. Low WHR or low BMI and high WHR, but not high BMI, are associated with all-cause mortality in advanced CKD.

## 1. Introduction

According to the World Health Organization, obesity has nearly tripled world widely since 1975 and has become an increasing health concern. High body mass index (BMI) was related to the development of kidney disease in general population [1]. A J-shaped relationship between body mass index (BMI) and all-cause-mortality was discovered in the general population [2,3]. Obesity and probably overweight are associated with increased mortality, in particular with vascular disease including ischemic heart diseases and stroke [4]. Surprisingly, high BMI is paradoxically linked with less mortality in dialysis patients, and this phenomenon is known as reverse epidemiology or the obesity paradox [5]. The reverse relationship between BMI and mortality has also been noted in patients with advanced chronic kidney disease (CKD) [6].

There are several hypotheses to explain the obesity paradox. First, central obesity or visceral fat, rather than BMI [7], could be a better indicator of obesity in many populations [8,9]. Central obesity measured by waist circumstance (WC) or waist-to-hip ratio (WHR) is less influenced by muscle and bone mass and is a predominant risk factor for metabolic syndrome [10]. In a large cohort of the general population, the association of WHR or WC with mortality was J-shaped before and linear after BMI adjustment [11,12]. One study has reported a sex difference, with higher WHR associated with mortality in women with congestive heart failure [13]. Thus, general and abdominal adiposity are both associated with the risk of death in the general population. Second, malnutrition–inflammation complex is more important than BMI in CKD. Malnutrition–inflammation complex shifts the nadir of the association between BMI and mortality to the right and changes the association from J- to U-shaped in some chronic diseases [4,14]. Malnutrition–inflammation complex does not change the nadir of central obesity in these chronic diseases. Thus, there is a BMI paradox but no central obesity paradox.

High WHR or WC is associated with mortality in the presence of BMI paradox in patients with CKD, especially those on dialysis. In dialysis patients, mortality increases linearly with WHR [15] or WC [15,16] and mortality decreases linearly as BMI increases. In other studies of dialysis patients, the mortality risk of high WHR [17] or WC [18] has been reported but not an association with BMI. In patients with stage 1–4 CKD and mean estimated glomerular filtration rate (eGFR) 50–70 mL/min/1.73 m^2^, high WHR [19] or WC [20] was associated with composite outcome or mortality, when BMI paradox was mild (high BMI had no or a small protective effect). exclusive reliance on BMI may underestimate the importance of obesity in patients with CKD. There are no reports about the risk of low WHR or WC in patients with CKD.

Few studies have demonstrated the association between WHR and mortality in patients with advanced CKD. Some studies have shown that central obesity is not an independent predictor of kidney disease progression or all-cause mortality [21]. We believe that BMI, central obesity, malnutrition–inflammation complex and sex difference should be all considered simultaneously in patients with advanced CKD. Under the practice of late dialysis initiation in Asia, malnutrition–inflammation complex is highly prevalent. The percentage of malnutrition–inflammation score (MIS) score ≥ 5 in our patients with stage 4–5 CKD with MIS ≥ 5 was similar to that in hemodialysis patients reported by Kalantar-Zadeh [22]. Racial difference and mean BMI < 25 kg/m^2^ in our patients with advanced CKD could also change the association between anthropometric measures and mortality [23]. Visceral fat has been demonstrated in some studies to be protective in animals and patients with cancer and cachexia [24,25]. We hypothesize that malnutrition–inflammation complex could also shift the nadir of WHR to the right and result in a J-shaped association between WHR and mortality. Thus, we would like to delineate the association of central obesity with mortality in patients with stage 3–5 CKD in the presence of reverse epidemiology.

## 2. Materials and Methods

### 2.1. Study Design and Participants

This prospective observational study, the Integrated CKD Care Program Kaohsiung for Delaying Dialysis, involving two affiliated hospitals of Kaohsiung Medical University in Southern Taiwan, was conducted from 11 November 2002 to 31 May 2009, as described previously [6]. We extended the follow-up period to 31 December 2014, compared with our previous study. The inclusion criterion was patients with stage 1–5 CKD not on renal replacement therapy (eGFR was calculated by Modification of Diet in Renal Disease [MDRD] equation). The exclusion criterion was acute kidney injury defined as >50% decrease in eGFR in 3 months. We included 3303 patients with stage 3–5 CKD.

To study the impact of BMI and WHR on mortality, participants were divided by these two parameters according to previous Asian BMI studies [26] and WHR quintiles. Patients with extreme BMI (<1% of participants), comprising seven with BMI < 14.9 kg/m^2^ and 58 with BMI > 35.1 kg/m^2^, were excluded as in our previous study [6]. Patients with stage CKD 1–2 were excluded in our study. We enrolled 3262 patients with stage 3–5 CKD and BMI 15.0–35.0 kg/m^2^. All patients provided informed consent to participate. The study protocol was approved by the Institutional Review Board of Kaohsiung Medical University Hospital.

### 2.2. Collection of Demographics, Medical and Laboratory Data

Baseline variables included demographic features (age and sex), medical history (diabetes mellitus, hypertension, cardiovascular disease [CVD], current smoking and cancer), examination findings (mean arterial pressure [MAP]), and laboratory data (albumin, hemoglobin, total cholesterol, C-reactive protein [CRP], glycated hemoglobin [HbA1c], bicarbonate, phosphate and urine protein-to-creatinine ratio [Upcr]). The demographic features were the baseline record and the medical history was obtained by doctors’ chart review and interview with patients. BMI was calculated as weight in kilograms divided by the square of height in meters. Waist and hip circumferences were measured according to WHO protocol [27]. Waist-to-hip ratio was defined as waist circumference (cm)/hip circumference (cm). Waist-to-height ratio was defined as waist circumference (cm)/height (cm). Conicity index was defined as waist circumference (cm)/0.109 × √ body weight (kilogram)/height (cm). The malnutrition-inflammation score (MIS) has 10 components with severity score from 0 (normal) to 3 (severely abnormal) in each component [28]. The 5 nutritional history–based components include body weight change, dietary intake, gastrointestinal symptoms, functional capacity, and comorbid conditions. We excluded dialysis vintage from the score and adopted the definition of score for comorbid condition from a CKD study [29]. The 2 physical examination components consist of assessment of subcutaneous body fat and signs of muscle wasting. Other 3 non-Subjective Global Assessment-based components are BMI, serum albumin level and serum total iron binding capacity. MIS >3 was associated with higher risk for mortality in previous CKD study [29]. The mean arterial pressure was calculated by the averaged systolic and diastolic blood pressure measured 3 months before and after enrollment using the formula one-third averaged systolic blood pressure plus two-thirds averaged diastolic pressure. Upcr was calculated as urine protein (mg) divided by urine creatinine (g) in random spot urine. Biochemistry measurements were done on screening visit, baseline visit and then every 3 months as per protocol. The laboratory data from 3 months before baseline to 3 months after were averaged and analyzed.

### 2.3. Outcomes

All-cause mortality was ascertained by review of death certificates using charts or the National Death Index. Models for the all-cause mortality included patients who reached renal replacement therapy and were censored only at death or the end of follow-up.

### 2.4. Statistical Analysis

Baseline characteristics of all patients and stratification by WHR were expressed as percentages for categorical data, mean ± standard deviation for continuous variables with approximately normal distribution, and median and interquartile range for continuous variables with skewed distribution. The differences between groups were checked by chi-square test for categorical variables or by one-way analysis of variance for continuous variables. Cox proportional hazards analysis was used to investigate the relationship of BMI and WHR with all-cause mortality. Skewed distributed continuous variables were log-transformed to attain normal distribution. Covariates were selected on the basis of clinical relevance, which was consistent with our previous paper [30]. The adjusted covariates included age, sex, eGFR, Upcr log, diabetes, cardiovascular disease, smoker, cancer, severe liver disease, hypertension, hemoglobin, cholesterol log, glycosylated hemoglobin, albumin, CRP ln and phosphorus. Statistical analysis was performed using SPSS for Windows version 20.0 (SPSS Inc., Chicago, IL, USA).

## 3. Results

### 3.1. Baseline Characteristics of Patients by WHR Quintiles

The baseline characteristics of the 3262 patients were divided into quintiles (Q1–5) according to WHR ratio (Table 1). The mean age was 63.5 ± 13.4 years, 1369 (42.0%) were female, 847 (26.0%) had cardiovascular disease, 2157 (66.1%) had hypertension, 1641 (50.3%) had diabetes, mean WC was 87.8 ± 13.3 cm, and average albumin level was 3.8 ± 0.5 g/dL. Overall BMI and eGFR were 24.6 ± 3.6 kg/m^2^ and 24.7 ± 15.1 mL/min/1.73 m^2^, respectively. The patients had an average number of metabolic syndrome components and Charlson Comorbidity Index of 3.1 ± 1.2 and 3.5 ± 2.1, respectively. The percentage of patients with diabetes, metabolic syndrome, cardiovascular disease and increased Charlson Comorbidity Index increased with WHR. There were 1290 (39.5%) patients with end-stage-renal disease (ESRD) and 900 (27.6%) had all-cause mortality.

### 3.2. Odds Ratios for Malnutrition–Inflammation Complex According to WHR

We reported the odds ratio (OR) for malnutrition–inflammation complex according with the fully adjusted Cox regression model grouped by WHR with adjustment for BMI. The reference groups were WHR Q2 in male patients and WHR Q3 in female patients. OR (95% confidence interval; CI) for malnutrition–inflammation complex was significantly increased in WHR Q1 (1.73; 1.13–2.64) male and Q1 (1.75; 1.06–2.87) female patients, respectively (Table 2). Adjustment for BMI had no effect on the result.

### 3.3. Association between BMI and All-Cause Mortality

**Figure 1:**
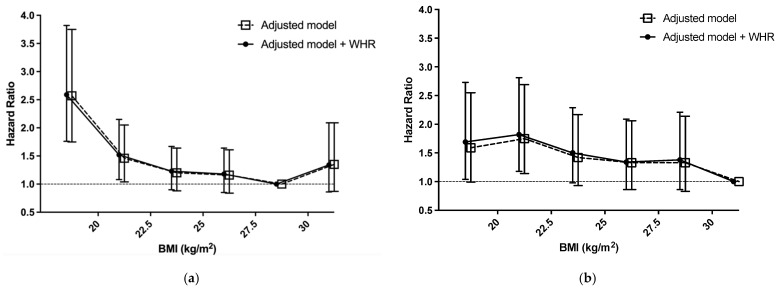
(**a**) HRs for mortality according to BMI in male patients before and after adjustment by WHR; (**b**) HRs for mortality according to BMI in female patients before and after adjustment by WHR. Abbreviations: BMI: body mass index, WHR: waist-to-hip ratio.

Our previous study showed that sex modified the association between BMI and all-cause mortality, so we separated the patients by sex. We analyzed the hazard ratio (HR) for mortality with the fully adjusted Cox regression model grouped by BMI with adjustment for WHR. The reference groups were BMI 27.6–30.0 kg/m^2^ in male patients and 30.1–35.0 kg/m^2^ in female patients. Mortality was significantly increased in male patients with BMI 15.0–20.0 kg/m^2^ (HR 2.59; 95% CI 1.76–3.82) and 20.1–22.5 kg/m^2^ (HR 1.52; 95% CI 1.08–2.15); and in female patients with BMI 15.0–20.0 kg/m^2^ (HR 1.69; 95% CI 1.04–2.73) and 20.1–22.5 kg/m^2^ (HR 1.82; 95% CI 1.18–2.81) (Table 3; Figure 1a,b). Adjustment for WHR had no effect on the result.

### 3.4. Association between WHR and Mortality

**Figure 2:**
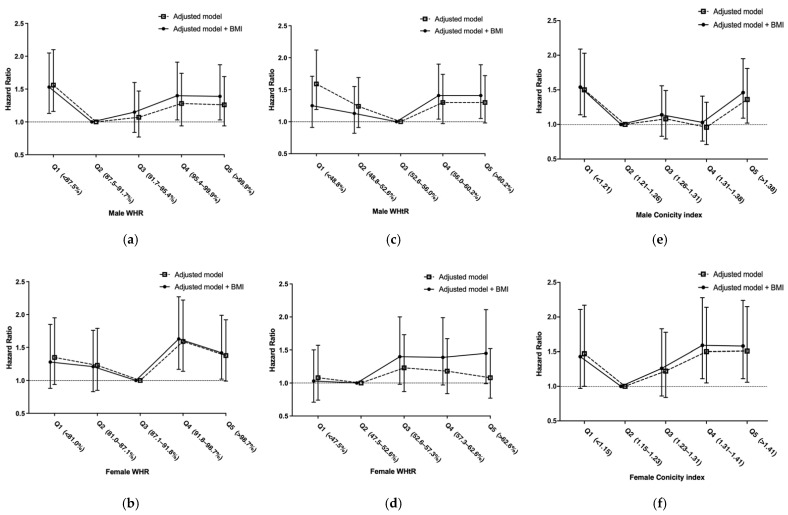
(**a**) HRs for mortality according to WHR in male patients before and after adjustment by BMI; (**b**) HRs for mortality according to WHR in female patients before and after adjustment by BMI; (**c**) HRs for mortality according to WHtR in male patients before and after adjustment by BMI; (**d**) HRs for mortality according to WHtR in female patients before and after adjustment by BMI; (**e**) HRs for mortality according to conicity index in male patients before and after adjustment by BMI; (**f**) HRs for mortality according to conicity index in female patients before and after adjustment by BMI. Abbreviations: BMI: body mass index, WHR: waist-to-hip ratio, WHtR: waist-to-height ratio, HHtR: waist-to-hip ratio.

To differentiate the effect of BMI and WHR, we reported the HR for mortality with the fully adjusted Cox regression model grouped by WHR with adjustment for BMI. The reference groups were WHR Q2 in male patients and WHR Q3 in female patients. Mortality was significantly increased in WHR Q4 (HR 1.40; 95% CI 1.03–1.91) and Q5 (HR 1.39; 95% CI 1.03–1.87) male patients; and significantly increased in WHR Q4 (HR 1.63; 95% CI 1.17–2.27) and Q5 (HR 1.42; 95% CI 1.02–1.99) female patients. There was a significant increase in mortality in WHR Q1 male patients (HR 1.53; 95% CI 1.13–2.05) (Table 4; Figure 2a,b). Adjustment for by BMI had no effect on the result. Sensitivity tests are shown in Supplementary Tables S1–S4.

### 3.5. Association between Waist-to-Height Ratio and Mortality

To differentiate the effect of BMI and waist-to-height ratio (WHtR), we reported the HR for mortality with the fully adjusted Cox regression model grouped by WHtR with adjustment for BMI. The reference groups were WHtR Q3 in male and WHtR Q2 in female patients. Mortality was significantly increased in WHtR Q4 (HR 1.41; 95% CI 1.04–1.90) and Q5 (HR 1.41; 95% CI 1.05–1.89) male patients; and marginally increased in WHtR Q3 (HR 1.40; 95% CI 0.98–2.0), Q4 (HR 1.39; 95% CI 0.97–1.99) and Q5 (HR 1.45; 95% CI 0.99–2.11) female patients (Figure 2c,d).

### 3.6. Association between Conicity Index and Mortality

To differentiate the effect of BMI and conicity index, we reported the HR for mortality with the fully adjusted Cox regression model grouped by conicity index with adjustment for BMI. The reference groups were conicity index Q2 in male and female patients. Mortality was significantly increased in conicity index Q1 male (HR 1.54; 95% CI 1.14–2.09), Q5 male (HR 1.46; 95% CI 1.09–1.95), Q4 female (HR 1.59; 95% CI 1.11–2.28) and Q5 female (HR 1.58; 95% CI 1.11–2.24) patients; and marginally increased in conicity index Q1 female patients (HR 1.43; 95% CI 0.97–2.11) (Figure 2e,f).

### 3.7. Association between BMI and WHR 

**Figure 3:**
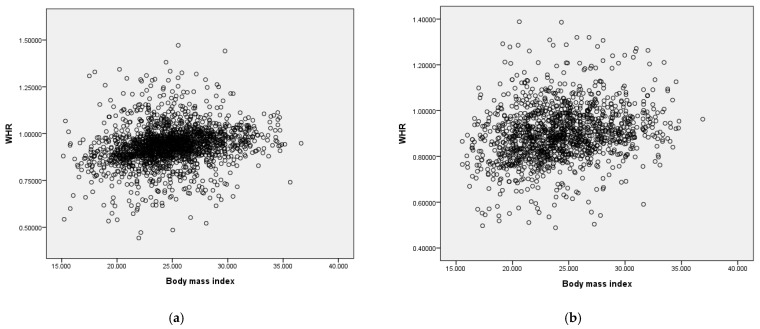
(**a**) Association between BMI and WHR in male patients (R^2^ = 0.044, Beta coefficient = 0.773); (**b**) Association between BMI and WHR in female patients (R^2^ = 0.055, Beta coefficient = 0.720). Abbreviations: BMI: body mass index, WHR: waist-to-hip ratio.

We analyzed the relationship of BMI and WHR in male and female patients by scatter plot. A low positive correlation was found between BMI and WHR in both male (R^2^ = 0.044, Beta coefficient = 0.773) and female (R^2^ = 0.055, Beta coefficient = 0.720) patients (Figure 3a,b).

### 3.8. The Association of Metabolic Syndrome Components and Inflammation with Mortality

**Figure 4:**
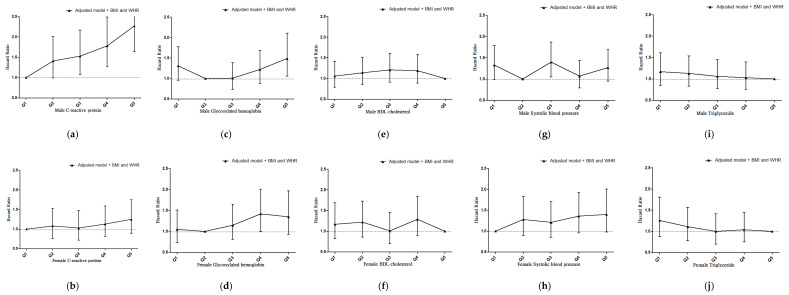
(**a**) HRs for mortality according to C-reactive protein in male patients after adjustment by BMI and WHR; (**b**) HRs for mortality according to C-reactive protein in female patients after adjustment by BMI and WHR; (**c**) HRs for mortality according to glycosylated hemoglobin in male patients after adjustment by BMI and WHR; (**d**) HRs for mortality according to glycosylated hemoglobin in female patients after adjustment by BMI and WHR; (**e**) HRs for mortality according to HDL-cholesterol in male patients after adjustment by BMI and WHR; (**f**) HRs for mortality according to HDL-cholesterol in female patients after adjustment by BMI and WHR; (**g**) HRs for mortality according to systolic blood pressure in male patients after adjustment by BMI and WHR; (**h**) HRs for mortality according to systolic blood pressure in female patients after adjustment by BMI and WHR; (**i**) HRs for mortality according to triglyceride in male patients after adjustment by BMI and WHR; (**j**) HRs for mortality according to triglyceride in female patients after adjustment by BMI and WHR. Adjusted model: adjusted for age, gender, eGFR, Upcr log, diabetes, cardiovascular disease, smoker, cancer, severe liver disease, hypertension, hemoglobin, cholesterol log, glycosylated hemoglobin, albumin, CRP ln and phosphorus. Abbreviations: BMI: body mass index, WHR: waist-to-hip ratio.

We reported the hazard ratio for mortality according with the fully adjusted Cox regression model grouped by metabolic syndrome components and inflammation with adjustment for BMI and WHR. The results showed a linear association between these factors with mortality regardless of statistical significance, except blood pressure (Figure 4 and Supplementary Table S5).

### 3.9. The Association between Malnutrition-Inflammation Score and Mortality

**Figure 5:**
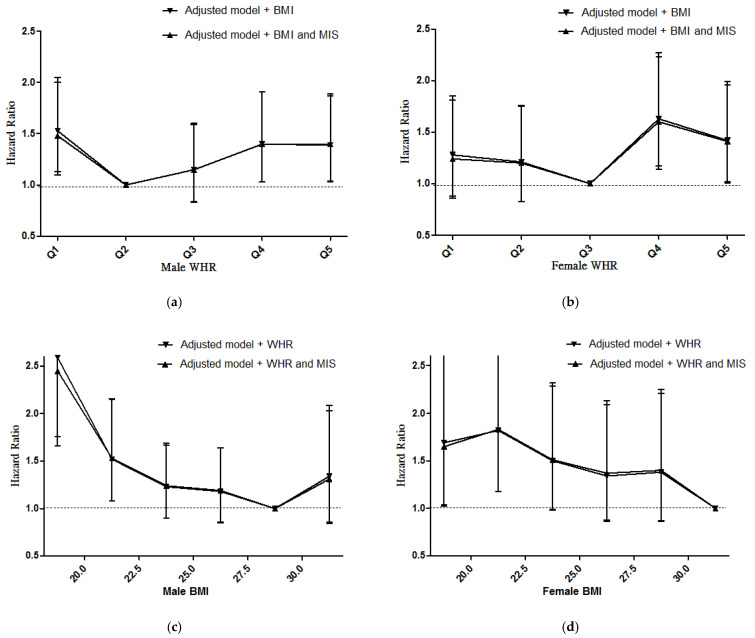
(**a**) HRs for mortality according to WHR in male patients before and after adjustment by BMI and MIS; (**b**) HRs for mortality according to WHR in female patients before and after adjustment by BMI and MIS; (**c**) HRs for mortality according to BMI in male patients before and after adjustment by WHR and MIS; (**d**) HRs for mortality according to BMI in female patients before and after adjustment by WHR and MIS. Adjusted model: adjusted for age, gender, eGFR, Upcr log, diabetes, cardiovascular disease, smoker, cancer, severe liver disease, hypertension, hemoglobin, cholesterol log, glycosylated hemoglobin, albumin, CRP ln and phosphorus. Abbreviations: BMI: body mass index, WHR: waist-to-hip ratio, MIS: Malnutrition-Inflammation Score.

We reported the hazard ratio for mortality according with the fully adjusted Cox regression model grouped by WHR and BMI with adjustment for MIS. The risk of low WHR or low BMI for all-cause mortality was not affected by including MIS in our model (Figure 5 and Supplementary Table S6).

## 4. Discussion

The present study showed that central obesity had different prognostic power compared to BMI in predicting all-cause mortality in patients with advance CKD. For mortality according to BMI, a reverse J-shaped relationship was found in male patients and a reverse association was found in female patients; however, for mortality according to central obesity (WHR, WHtR and conicity index), a U-shaped relationship was found in both sexes. Our study included 3262 Asian patients with stage 3–5 CKD, and the average BMI was 24.6 ± 3.6 kg/m^2^ (15.0–35.0 kg/m^2^) with average WC of 87.8 ± 13.3 cm. The obesity paradox was seen in female patients with high BMI, but high central obesity still predicted mortality. Pischon et al. suggested the use of WC or WHR in addition to BMI in assessing mortality risk [11], and accordingly, WC with normalization by hip and height was used as the anthropometric measure for central obesity in our study. In male patients, we found that high BMI and high central obesity were positively associated with increased all-cause mortality, but central obesity, especially WHR, had a different prognostic power. Central obesity showed increased mortality in male patients with WHR Q4 (HR 1.40; 95% CI 1.03–1.91) and WHR Q5 (HR 1.39; 95% CI 1.03–1.87), which included 40% of the population. Increased mortality (HR 1.34; 95% CI 0.86–2.09) was only found in 136 male patients with BMI 30.1–35.0 kg/m^2^, which comprised < 10% of the total male population of 1893.

In the general population, BMI is a strong predictor for overall mortality. The relationship between BMI and all-cause mortality was discovered to be J-shaped in a large analysis of prospective studies including 1.46 million white adults [2], and U-shaped in a pooled analysis of 850,000 East Asians [26]. Compared with Europeans, the risk of death in the Asian population seems to be strongly affected by low rather than high BMI and the lowest risk of death was seen in those with BMI 22.6–27.5 kg/m^2^ [26]. BMI showed a U-shaped association with death from overall cardiovascular disease among 835,082 East Asians [31]. However, the effect of overweight and obesity in patients with CKD undergoing maintenance hemodialysis is paradoxically associated with decreased cardiovascular diseases and all-cause mortality, leading to improved survival recognized as reverse epidemiology [5]. The inverse relationship of BMI and mortality was also found in patients with advanced-stage CKD [32] and multiple hypothesis for reverse epidemiology were raised and discussed.

Emerging evidence shows that central obesity is different from BMI in predicting mortality of patients with advanced CKD. This is due to the possibility that BMI reflects obesity caused by muscle mass and water retention as well as fat. Central obesity (measured by WHR and WHtR) is a marker of dysfunction of adipose tissue and the most prevalent manifestation of metabolic syndrome [10]. Metabolic syndrome is well recognized and associated with increased diabetes and cardiovascular disease [33]. Metabolic syndrome components and inflammation are important factors associated with all-cause mortality in obese CKD patients. Our results showed that these factors were linearly associated with mortality regardless of statistical significance, except blood pressure. It is suggested that mortality increases linearly with central obesity in dialysis patients. Postorino et al. [15] presented a prospective cohort study of 537 patients with ESRD, which showed a significant relationship between WC and WHR with all-cause mortality. Kim et al. [16] obtained the Korean National Health Insurance database of 18,699 participants undergoing hemodialysis and showed that all-cause mortality was positively associated with WC.

Our results confirmed a U-shaped association between WHR and all-cause mortality in both sexes. A significant increase in mortality was noticed in WHR Q4 and Q5 of both sexes compared with reference groups (WHR Q2 in male and WHR Q3 in female patients). The result is consistent with other central obesity studies that showed increased mortality as WHR increased. However, in low WHR quintiles, there was an increase trend in all-cause mortality in both sexes. The difference between our study and others may be due to different baseline body composition. The MIS is strongly linked with nutritional status assessment in patients with CKD [34]. Due to late initiation of dialysis in Asia, malnutrition–inflammation complex is highly prevalent. Our study showed that low WHR is associated with high MIS, which shifted the nadir of WHR to the right and result in a J-shaped association between WHR and mortality. Malnutrition-inflammation was noticed with increased mortality [35]. MIS is a useful tool to assess the protein energy wasting (PEW) score in nondialyzed CKD patients and identified those at increased mortality risk [29]. The PEW score reflects systemic inflammation, malnutrition and wasting among CKD and dialysis patients, and is associated with adverse clinical outcomes [36,37]. Our result showed that low WHR or low BMI was associated MIS (Supplementary Table S7). However, the risk of low WHR or low BMI for all-cause mortality was not affected by including MIS in the model. The high risk of low WHR or low BMI for all-cause mortality could not be totally explained by MIS. Recent studies demonstrated an “adipokine paradox”, for example leptin secreted by the adipocyte might be cardioprotection [38], further studies should be done. For low WHR, increased mortality was noticed significantly in WHR Q1 male patients but not in female patients. Sex difference was noticed in previous studies of WHR and mortality in patients with congestive heart failure [13], and this may have been due to the protection of sex hormones [39]. In female patients with low WHR, estrogen has a significant influence on cardiovascular events in the presence of malnutrition and atherosclerosis.

In patients with advance CKD, central obesity is linearly associated with all-cause mortality. Elsayed et al. [19] revealed enhancement of cardiac events as WHR increased in a cohort study of 1669 patients with CKD. Kramer et al. [20] analyzed 5805 adults in the REGARDS (Reasons for Geographic and Racial Differences in Stroke) Study and concluded that each 1-cm increase in WC was associated with a 2% increase in mortality risk. However, these studies dealt with overweight and obese patients (both excluded individuals with BMI < 18.5 kg/m^2^) and do not represent the general population comprehensively. Some studies have even shown that central obesity is not associated with kidney disease progression or all-cause mortality [21].

We also tested for anthropometric markers of central obesity, including WHtR and conicity index. WHtR is a simple measure and has similar reference values for both sexes [40] and is an indicator of cardiometabolic risk [41]. Conicity index is one of the most accurate measures for discrimination of visceral obesity [40] and is a good predictor of cardiovascular risk [37], especially in male patients. Our result showed that the associations between the two indexes and all-cause mortality were U-shaped in men and women. These results were consistent with literatures. Welborn et al. [42] observed 9309 Australian adults and suggested that WHR was superior than WHtR and WC in predicting all-cause mortality and CVD mortality. Data from the Jackson Heart Study with 3976 African American participants [43] suggested that WHR is an important anthropometric measure to analyze obesity-related risk for mortality. Sex difference was also mentioned in a prospective cohort study [44], which showed that WHtR was associated with increased mortality only in the male population.

The main strength of the present study was the large sample size (*n* = 3262) and enrollment of patients with stage 3–5 CKD with BMI 15.0–35.0 kg/m^2^, which covers underweight, normal-weight and overweight patients. There were several limitations to the present study. First, we used baseline anthropometric measurements for analysis and time-dependent change was not obtained. Second, the Integrated CKD Care Program Kaohsiung for Delaying Dialysis is a sample of the Asian population, and we were not able to address whether race was important in body composition or outcome. Third, dietary and medication factors were lacking in our study, and we need to bear in mind the importance of their effects on obesity and CKD incidence. Fourth, the study participants had advanced-stage CKD, thus the results may not be generalized to all CKD populations. More studies of different central obesity markers (including WHR, WC and WHtR) are needed to clarify the best predictor for all-cause mortality in patients with advanced CKD.

## 5. Conclusions

Our study showed a U-shaped association between central obesity and all-cause mortality in both sexes. Low BMI and low WHR are associated with all-cause mortality, while high WHR, but not high BMI, is associated with all-cause mortality in patients with advanced CKD.

## Figures and Tables

**Table 1:** Baseline characteristics of patients by WHR quintiles.

		Waist-to-Hip Ratio					
Variable	All	Q1	Q2	Q3	Q4	Q5	*p* Value (ANOVA)
WHR (male) (%)		<87.5	87.5–91.7	91.7–95.4	95.4–99.9	>99.9	
WHR (female) (%)		<81.0	81.0–87.1	87.1–91.8	91.8–98.7	>98.7	
No. of patients	3262	651 (20.0%)	653 (20.0%)	653 (20.0%)	653 (20.0%)	652 (20.0%)	
Demographics and medical history							
Age (years)	63.5 (13.4)	58.4 (15.2)	62.1 (12.8)	63.5 (12.6)	66.1 (12.2)	67.5 (12.3)	<0.001
Sex (female)	1369 (42.0%)	273 (41.9%)	274 (42.0%)	274 (42.0%)	274 (42.0%)	274 (42.0%)	1.000
Comorbidity							
Cardiovascular disease	847 (26.0%)	135 (20.7%)	145 (22.2%)	162 (24.8%)	186 (28.5%)	219 (33.5%)	<0.001
Ischemic heart disease	485 (14.9%)	73 (11.2%)	95 (14.5%)	92 (14.1%)	111 (17.0%)	114 (17.5%)	0.011
Congestive heart disease	407 (12.5%)	63 (9.7%)	63 (9.6%)	72 (11.0%)	92 (14.1%)	117 (17.9%)	<0.001
Cerebrovascular disease	554 (17.0%)	89 (13.7%)	93 (14.2%)	109 (16.7%)	125 (19.1%)	138 (21.1%)	<0.001
Hypertension	2157 (66.1%)	410 (63.0%)	432 (66.2%)	444 (68.0%)	429 (65.7%)	442 (67.7%)	0.324
Diabetes mellitus	1641 (50.35%)	278 (42.7%)	276 (42.3%)	320 (49.0%)	356 (54.5%)	411 (62.9%)	<0.001
Hyperuricemia	558 (17.1%)	99 (15.2%)	94 (14.4%)	127 (19.4%)	122 (18.7%)	116 (17.8%)	0.063
Severe liver disease	160 (4.9%)	34 (5.2%)	31 (4.7%)	28 (4.3%)	37 (5.7%)	30 (4.6%)	0.799
Cancer	289 (8.9%)	53 (8.1%)	68 (10.4%)	64 (9.8%)	50 (7.7%)	54 (8.3%)	0.338
Charlson score	3.5 (2.1)	3.3 (1.9)	3.4 (2.0)	3.5 (2.1)	3.6 (2.1)	3.8 (2.1)	<0.001
Metabolic syndrome *	3.1 (1.2)	2.3 (1.1)	2.8 (1.2)	3.3 (1.2)	3.5 (1.1)	3.7 (1.0)	<0.001
Mean BP (mmHg)	99.9 (13.7)	98.4 (14.1)	99.2 (13.3)	100.8 (14.2)	100.3 (13.6)	100.7 (13.1)	0.004
Body mass index (kg/m^2^)	24.6 (3.6)	22.9 (3.5)	24.0 (3.3)	25.0 (3.4)	25.4 (3.6)	25.7 (3.6)	<0.001
Waist (cm)	87.8 (13.3)	72.2 (10.0)	83.2 (7.3)	88.1 (7.6)	92.8 (7.5)	102.7 (10.4)	
Laboratory data							
eGFR (mL/min/1.73 m^2^)	24.7 (15.1)	22.8 (15.6)	26.1 (15.3)	26.1 (15.1)	26.6 (15.1)	22.1 (13.8)	<0.001
UPCR (mg/g)	1110 (405–2542)	1192 (447–2899)	969 (344–2182)	970 (377–2239)	1107 (394–2371)	1461 (530–3118)	<0.001
Hemoglobin (g/dL)	10.9 (2.3)	10.5 (2.2)	11.1 (2.3)	11.3 (2.4)	11.2 (2.4)	10.6 (2.3)	<0.001
Albumin (g/dL)	3.8 (0.5)	3.8 (0.6)	3.9 (0.5)	3.9 (0.5)	3.8 (0.5)	3.7 (0.5)	<0.001
GPT (U/L)	24.6 (24.1)	23.8 (20.9)	24.7 (30.0)	26.0 (27.9)	24.9 (21.7)	23.8 (17.5)	0.406
Total cholesterol (mg/dL)	191 (162–222)	190 (160–217)	192 (166–224)	191 (162–223)	192 (163–224)	190 (160–221)	0.609
Triglyceride (mg/dL)	126 (91–184)	111 (81–158)	123 (86–179)	137 (96–194)	131 (95–194)	133 (98–191)	<0.001
HDL-cholesterol (mg/dL)	42.4 (13.7)	45.2 (15.9)	43.5 (14.0)	42.0 (13.4)	41.3 (12.6)	40.3 (11.9)	<0.001
LDL-cholesterol (mg/dL)	113.1 (38.7)	114.4 (41.3)	113.8 (38.4)	112.1 (37.4)	113.8 (39.1)	111.4 (37.3)	0.580
C-reactive protein (mg/L)	1.2 (0.4–5.2)	1.2 (0.4–5.1)	0.9 (0.3–2.8)	0.9 (0.3–3.9)	1.2 (0.5–5.7)	2.3 (0.5–10.5)	<0.001
Sodium (mEq/L)	138.2 (3.7)	138.0 (3.7)	138.3 (3.4)	138.3 (3.4)	138.2 (3.7)	137.9 (4.2)	0.014
Potassium (mEq/L)	4.4 (0.6)	4.3 (0.6)	4.3 (0.6)	4.3 (0.6)	4.4 (0.6)	4.4 (0.6)	0.165
Calcium (mg/dL)	9.1 (0.8)	9.0 (0.8)	9.1 (0.7)	9.1 (0.8)	9.1 (0.8)	9.1 (0.9)	<0.001
Glycosylated hemoglobin (%)	6.5 (1.6)	6.3 (1.5)	6.2 (1.4)	6.5(1.6)	6.7(1.7)	6.7 (1.7)	<0.001
Uric acid (mg/dL)	7.9 (1.9)	7.8 (2.0)	7.8 (2.0)	7.9 (1.8)	7.9 (1.9)	8.0 (2.0)	0.139
Outcomes							
ESRD	1290 (39.5%)	203 (31.2%)	304 (46.6%)	287 (44.0%)	281 (43.0%)	215 (32.9%)	<0.001
All-cause mortality	900 (27.6%)	183 (28.1%)	128 (19.6%)	138 (21.1%)	198 (30.3%)	253 (38.7%)	<0.001

Data are presented as mean (standard error), median (interquartile range), or count (percentage %). * Components of metabolic syndrome: 1. waist circumference ≥ 90 cm in men or ≥ 80 cm in women; 2. systolic blood pressure ≥ 130 mmHg or diastolic blood pressure ≥ 85 mmHg or hypertension; 3. HDL cholesterol > 40 mg/dL in men or > 50 mg/dL in women; 4. triglycerides ≥ 150 mg/dL; and 5. fasting blood glucose ≥ 100 mg/dL or diabetes mellitus. Abbreviations: WHR: waist-to-hip ratio, BP: blood pressure, eGFR: estimated glomerular filtration rate, UPCR: urine protein and creatinine ratio, GPT: glutamic pyruvic transaminase, LDL: low-density lipoprotein, ESRD: end-stage renal disease.

**Table 2:** Odds ratios for malnutrition–inflammation complex according to WHR.

OR for Malnutrition- Inflammation		Waist-to-Hip Ratio				
		Q1	Q2	Q3	Q4	Q5
Male	Adjusted model	2.07 (1.38–3.12) *	1 (reference)	0.96 (0.64–1.43)	0.98 (0.65–1.46)	0.98 (0.65–1.46)
	Adjusted model + BMI	1.73 (1.13–2.64) *	1 (reference)	1.05 (0.70–1.58)	1.06 (0.70–1.60)	1.01 (0.66–1.56)
Female	Adjusted model	2.22 (1.39–3.54) *	1.39 (0.89–2.18)	1 (reference)	1.14 (0.73–1.79)	1.14 (0.73–1.79)
	Adjusted model + BMI	1.75 (1.06–2.87) *	1.38 (0.86–2.20)	1 (reference)	1.16 (0.72–1.86)	1.03 (0.63–1.69)

Values expressed as hazard ratio (HR) and 95% confidence interval (CI). Adjusted model: adjusted for age, sex, eGFR, Upcr log, diabetes, cardiovascular disease, smoker, cancer, severe liver disease, hypertension, hemoglobin, cholesterol log, glycosylated hemoglobin, albumin, CRP ln and phosphorus. * *p* < 0.05 compared with reference WHR category. Abbreviations: BMI: body mass index, HR: hazard ratio, WHR: waist-to-hip ratio, eGFR: estimated glomerular filtration rate, Upcr: urine protein and creatinine ratio, CRP: C-reactive protein.

**Table 3:** HRs for mortality according to BMI.

HR for Mortality		BMI (kg/m^2^)					
		15.0–20.0	20.1–22.5	22.6–25.0	25.1–27.5	27.6–30.0	30.1–35.0
Male	Adjusted model	2.57 (1.75–3.75) ^†^	1.46 (1.04–2.05) *	1.20 (0.88–1.64)	1.16 (0.84–1.61)	1 (reference)	1.35 (0.87–2.09)
	Adjusted model + WHR	2.59 (1.76–3.82) ^†^	1.52 (1.08–2.15) *	1.23 (0.90–1.67)	1.18 (0.85–1.64)	1 (reference)	1.34 (0.86–2.09)
Female	Adjusted model	1.59 (0.99–2.55)	1.75 (1.14–2.69) *	1.42 (0.93–2.17)	1.33 (0.86–2.06)	1.33 (0.83–2.14)	1 (reference)
	Adjusted model + WHR	1.69 (1.04–2.73) *	1.82 (1.18–2.81) *	1.50 (0.98–2.29)	1.34 (0.86–2.09)	1.38 (0.86–2.21)	1 (reference)

Values expressed as hazard ratio (HR) and 95% confidence interval (CI). Adjusted model: adjusted for age, sex, eGFR, Upcr log, diabetes, cardiovascular disease, smoker, cancer, severe liver disease, hypertension, hemoglobin, cholesterol log, glycosylated hemoglobin, albumin, CRP ln and phosphorus. * *p* < 0.05 compared with reference BMI category. ^†^ *p* < 0.001 compared with reference BMI category. Abbreviations: BMI: body mass index, HR: hazard ratio, WHR: waist-to-hip ratio, eGFR: estimated glomerular filtration rate, Upcr: urine protein and creatinine ratio, CRP: C-reactive protein.

**Table 4:** HRs for mortality according to WHR.

HR for Mortality		Waist-to-Hip Ratio				
		Q1	Q2	Q3	Q4	Q5
Male	Adjusted model	1.56 (1.16–2.10) *	1 (reference)	1.07 (0.77–1.47)	1.28 (0.94–1.74)	1.26 (0.94–1.69)
	Adjusted model + BMI	1.53 (1.13–2.05) *	1 (reference)	1.15 (0.84–1.60)	1.40 (1.03–1.91) *	1.39 (1.03–1.87) *
Female	Adjusted model	1.35 (0.94–1.95)	1.23 (0.85–1.79)	1 (reference)	1.59 (1.14–2.22) *	1.38 (0.99–1.92)
	Adjusted model + BMI	1.28 (0.88–1.85)	1.21 (0.83–1.76)	1 (reference)	1.63 (1.17–2.27) *	1.42 (1.02–1.99) *

Values expressed as hazard ratio (HR) and 95% confidence interval (CI). Adjusted model: adjusted for age, sex, eGFR, Upcr log, diabetes, cardiovascular disease, smoker, cancer, severe liver disease, hypertension, hemoglobin, cholesterol log, glycosylated hemoglobin, albumin, CRP ln and phosphorus. * *p* < 0.05 compared with reference WHR category. Abbreviations: BMI: body mass index, HR: hazard ratio, WHR: waist-to-hip ratio, eGFR: estimated glomerular filtration rate, Upcr: urine protein and creatinine ratio, CRP: C-reactive protein.

## Data Availability

Study data are available from the corresponding author (C.-C.H.) upon request.

## Supplementary Materials

The following are available online at https://www.mdpi.com/article/10.3390/jpm11121355/s1, Supplementary Table S1: Multivariate linear regression for waist-to-hip ratio (per 0.05 increase), Supplementary Table S2: Hazard ratios for mortality according to waist-to-height ratio, Supplementary Table S3: Hazard ratios for mortality according to waist circumference, Supplementary Table S4: Hazard ratios for mortality according to hip-to-height ratio, Supplementary Table S5: Hazard ratios for mortality according to metabolic syndrome components and inflammation, Supplementary Table S6: Hazard ratios for mortality according to malnutrition-inflammation syndrome, Supplementary Table S7: Odds ratios for malnutrition-inflammation according to body mass index and waist-to-hip ratio.
