# Supplementary material for: U-Shaped Association between Waist-to-Hip Ratio and All-Cause Mortality in Stage 3–5 Chronic Kidney Disease Patients with Body Mass Index Paradox
Source: J Pers Med. 2021 Dec 13;11(12):1355. doi: 10.3390/jpm11121355 (PMC8703404; doi:10.3390/jpm11121355)
Supplement: Supplementary file 1 [file jpm-11-01355-s001.zip › Supplementary tables 1-7.pdf]

Supplement Table S1. Multivariate linear regression for waist-to-hip ratio (per 0.05 increase)

	Beta coefficient	95% CI Beta coefficient	P value
Constant	13.213		
Age (years)	0.034	0.028 to 0.040	<0.001
Gender (female vs male)	-0.675	-0.845 to -0.505	<0.001
eGFR (ml/min/1.73 m^2)	-0.004	-0.012 to 0.003	0.278
Upcr log	0.243	0.062 to 0.424	0.008
Diabetes mellitus	0.245	0.056 to 0.434	0.011
Cardiovascular disease	0.120	-0.061 to 0.301	0.194
Smoker	0.119	-0.123 to 0.362	0.335
Cancer	-0.113	-0.377 to 0.152	0.404
Severe liver disease	-0.115	-0.460 to 0.230	0.513
Hypertension	-0.106	-0.271 to 0.060	0.210
Hemoglobin (g/dl)	0.004	-0.046 to 0.054	0.869
Body mass index (kg/m^2)	0.135	0.114 to 0.157	<0.001
Cholesterol log	-0.150	-0.858 to 0.558	0.678
Glycosylated hemoglobin (%)	0.029	-0.029 to 0.086	0.332
Albumin (g/dl)	0.037	-0.134 to 0.208	0.669
CRP ln	0.095	0.007 to 0.183	0.034
Phosphorus (mg/dl)	-0.025	-0.102 to 0.051	0.516

Abbreviations: WHR: waist-to-hip ratio, CI: confidence interval, eGFR: estimated glomerular filtration rate, Upcr: urine protein and creatinine ratio, CRP: C-reactive protein.

Supplement Table S2. Hazard ratios for mortality according to waist-to-height ratio

HR for mortality		Waist-to-height ratio				
		Q1	Q2	Q3	Q4	Q5
	WHTR (%) Male	<48.8	48.8-52.6	52.6-56	56-60.2	>60.2
	WHTR (%) Female	<47.5	47.5-52.6	52.6-57.3	57.3-62.6	>62.6
Male	Adjusted model	1.59 (1.19-2.12)*	1.24 (0.91-1.69)	1 (reference)	1.30 (0.97-1.74)	1.30 (0.98-1.72)
	Adjusted model + BMI	1.25 (0.91-1.71)	1.13 (0.82-1.55)	1 (reference)	1.41 (1.04-1.90)*	1.41 (1.05-1.89)*
Female	Adjusted model	1.08 (0.74-1.57)	1 (reference)	1.23 (0.87-1.73)	1.18 (0.84-1.67)	1.08 (0.77-1.52)
	Adjusted model + BMI	1.03 (0.71-1.50)	1 (reference)	1.40 (0.98-2.00)	1.39 (0.97-1.99)	1.45 (0.99-2.11)

Values expressed as hazard ratio (HR) and 95% confidence interval (CI).

Adjusted model: adjusted for age, gender, eGFR, Upcr log, diabetes, cardiovascular disease, smoker, cancer, severe liver disease, hypertension, hemoglobin, cholesterol log, glycosylated hemoglobin, albumin, CRP ln and phosphorus.

$^{*}P < 0.05$ compared with reference WHTR category.

Abbreviations: HR: hazard ratio, WHTR: waist-to-height ratio, BMI: Body mass index, eGFR: estimated glomerular filtration rate, Upcr: urine protein and creatinine ratio, CRP: C-reactive protein.

Supplement Table S3. Hazard ratios for mortality according to waist circumference

HR for mortality		Waist circumference				
		Q1	Q2	Q3	Q4	Q5
Male	Adjusted model	1.52 (1.15-2.01) *	1.15 (0.86-1.54)	1.26 (0.94-1.68)	1 (reference)	1.26 (0.96-1.66)
	Adjusted model + BMI	1.15 (0.84-1.57)	1.00 (0.73-1.37)	1.19 (0.89-1.60)	1 (reference)	1.25 (0.94-1.66)
Female	Adjusted model	1.06 (0.74-1.51)	1 (reference)	1.25 (0.90-1.75)	0.98 (0.69-1.37)	1.00 (0.72-1.40)
	Adjusted model + BMI	1.01 (0.70-1.46)	1 (reference)	1.39 (0.99-1.96)	1.11 (0.78-1.59)	1.29 (0.89-1.86)

Values expressed as hazard ratio (HR) and 95% confidence interval (CI).

Adjusted model: adjusted for age, gender, eGFR, Upcr log, diabetes, cardiovascular disease, smoker, cancer, severe liver disease, hypertension, hemoglobin,

cholesterol log, glycosylated hemoglobin, albumin, CRP ln and phosphorus.

*$P < 0.05$ compared with reference WC category.

Abbreviations: BMI: Body mass index, HR: hazard ratio, WC: waist circumference, eGFR: estimated glomerular filtration rate, Upcr: urine protein and creatinine ratio, CRP: C-reactive protein.

Supplement Table S4. Hazard ratios for mortality according to hip-to-height ratio

HR for mortality		Hip-to-height ratio				
		Q1	Q2	Q3	Q4	Q5
Male	Adjusted model	1 (reference)	0.99 (0.75-1.31)	0.87 (0.66-1.13)	0.83 (0.62-1.09)	0.85 (0.65-1.11)
	Adjusted model + BMI	1 (reference)	1.25 (0.93-1.68)	1.23 (0.90-1.67)	1.22 (0.87-1.70)	1.34 (0.93-1.92)
Female	Adjusted model	1 (reference)	1.02 (0.73-1.44)	1.07 (0.77-1.49)	1.17 (0.84-1.62)	0.86 (0.62-1.21)
	Adjusted model + BMI	1 (reference)	1.10 (0.77-1.56)	1.27 (0.88-1.83)	1.51 (1.03-2.22)*	1.30 (0.83-2.04)

Values expressed as hazard ratio (HR) and 95% confidence interval (CI).

Adjusted model: adjusted for age, gender, eGFR, Upcr log, diabetes, cardiovascular disease, smoker, cancer, severe liver disease, hypertension, hemoglobin, cholesterol log, glycosylated hemoglobin, albumin, CRP ln and phosphorus.

*$P < 0.05$ compared with reference HHR category.

Abbreviations: BMI: Body mass index, HR: hazard ratio, WHR: waist-to-hip ratio, eGFR: estimated glomerular filtration rate, Upcr: urine protein and creatinine ratio, CRP: C-reactive protein

Supplement Table S5. Hazard ratios for mortality according to metabolic syndrome components and inflammation

HR for mortality		C-reactive protein				
		Q1	Q2	Q3	Q4	Q5
Male	Adjusted model	1 (reference)	1.62 (1.14-2.30)*	1.60 (1.13-2.26)*	2.18 (1.57-3.03)**	2.69 (1.95-3.70)**
	Adjusted model + BMI and WHR	1 (reference)	1.41 (0.99-2.01)	1.53 (1.08-2.17)*	1.78 (1.28-2.49)**	2.27 (1.64-3.14)**
Female	Adjusted model	1 (reference)	1.12 (0.79-1.57)	1.00 (0.70-1.43)	1.18 (0.84-1.64)	1.31 (0.94-1.82)
	Adjusted model + BMI and WHR	1 (reference)	1.08 (0.76-1.53)	1.03 (0.72-1.48)	1.13 (0.81-1.59)	1.25 (0.89-1.75)

	HR for mortality	Glycosylated hemoglobin				
		Q1	Q2	Q3	Q4	Q5
Male	Adjusted model	1.41 (1.04-1.92)*	1 (reference)	0.98 (0.71-1.34)	1.23 (0.89-1.70)	1.53 (1.09-2.15)*
	Adjusted model + BMI and WHR	1.31 (0.96-1.78)	1 (reference)	1.01 (0.73-1.39)	1.22 (0.88-1.69)	1.49 (1.06-2.11)*
Female	Adjusted model	1.10 (0.77-1.56)	1 (reference)	1.10 (0.78-1.56)	1.42 (1.00-2.00)	1.34 (0.93-1.95)
	Adjusted model + BMI and WHR	1.05 (0.74-1.51)	1 (reference)	1.15 (0.81-1.65)	1.42 (1.00-2.01)	1.35 (0.93-1.97)
	HR for mortality	HDL-cholesterol				
		Q1	Q2	Q3	Q4	Q5
Male	Adjusted model	0.94 (0.71-1.24)	0.99 (0.75-1.30)	1.07 (0.81-1.41)	1.09 (0.82-1.44)	1 (reference)
	Adjusted model + BMI and WHR	1.06 (0.79-1.42)	1.14 (0.86-1.52)	1.21 (0.91-1.61)	1.19 (0.89-1.58)	1 (reference)
Female	Adjusted model	1.27 (0.90-1.78)	1.28 (0.91-1.80)	0.95 (0.66-1.37)	1.22 (0.85-1.73)	1 (reference)
	Adjusted model + BMI and WHR	1.17 (0.82-1.69)	1.22 (0.86-1.73)	1.01 (0.70-1.46)	1.29 (0.90-1.84)	1 (reference)
	HR for mortality	Systolic blood pressure				
		Q1	Q2	Q3	Q4	Q5
Male	Adjusted model	1.52 (1.13-2.04)*	1 (reference)	1.41 (1.06-1.88)*	1.02 (0.76-1.36)	1.36 (1.02-1.81)*
	Adjusted model + BMI and WHR	1.33 (0.99-1.79)	1 (reference)	1.40 (1.05-1.87)*	1.07 (0.79-1.44)	1.27 (0.95-1.69)
Female	Adjusted model	1 (reference)	1.15 (0.81-1.64)	1.08 (0.77-1.53)	1.17 (0.83-1.64)	1.30 (0.92-1.83)
	Adjusted model + BMI and WHR	1 (reference)	1.28 (0.90-1.83)	1.21 (0.85-1.71)	1.36 (0.96-1.93)	1.40 (0.98-2.01)
	HR for mortality	Triglyceride				
		Q1	Q2	Q3	Q4	Q5
Male	Adjusted model	1.42 (1.06-1.89)*	1.26 (0.94-1.68)	1.18 (0.88-1.58)	1.09 (0.82-1.47)	1 (reference)
	Adjusted model + BMI and WHR	1.17 (0.85-1.61)	1.13 (0.83-1.54)	1.06 (0.78-1.45)	1.03 (0.76-1.40)	1 (reference)
Female	Adjusted model	1.40 (1.00-1.96)*	1.09 (0.79-1.51)	0.98 (0.70-1.38)	1.04 (0.75-1.43)	1 (reference)
	Adjusted model + BMI and WHR	1.26 (0.88-1.81)	1.11 (0.78-1.57)	1.00 (0.70-1.42)	1.04 (0.75-1.45)	1 (reference)

Values expressed as hazard ratio (HR) and 95% confidence interval (CI).

Adjusted model: adjusted for age, gender, eGFR, Upcr log, diabetes, cardiovascular disease, smoker, cancer, severe liver disease, hypertension, hemoglobin, cholesterol log, glycosylated hemoglobin, albumin, CRP ln and phosphorus.

*$P < 0.05$ compared with reference metabolic syndrome component category.

**P < 0.001 compared with reference metabolic syndrome component category.

Abbreviations: HR: hazard ratio, BMI: Body mass index, WHR: Waist-to-hip ratio, eGFR: estimated glomerular filtration rate, Upcr: urine protein and creatinine ratio, CRP: C-reactive protein.

Supplement Table S6. Hazard ratios for mortality according to malnutrition-inflammation syndrome

HR for mortality		Waist-to-hip ratio				
		Q1	Q2	Q3	Q4	Q5
Male	Adjusted model	1.56 (1.16-2.10)*	1 (reference)	1.07 (0.77-1.47)	1.28 (0.94-1.74)	1.26 (0.94-1.69)
	Adjusted model + BMI	1.53 (1.13-2.05)*	1 (reference)	1.15 (0.84-1.60)	1.40 (1.03-1.91)*	1.39 (1.03-1.87)*
	Adjusted model + BMI and MIS	1.48 (1.10-2.00)*	1 (reference)	1.15 (0.83-1.59)	1.40 (1.03-1.91)*	1.40 (1.04-1.89)*
Female	Adjusted model	1.35 (0.94-1.95)	1.23 (0.85-1.79)	1 (reference)	1.59 (1.14-2.22)*	1.38 (0.99-1.92)
	Adjusted model + BMI	1.28 (0.88-1.85)	1.21 (0.83-1.76)	1 (reference)	1.63 (1.17-2.27)*	1.42 (1.02-1.99)*
	Adjusted model + BMI and MIS	1.24 (0.86-1.81)	1.20 (0.83-1.75)	1 (reference)	1.60 (1.14-2.23)*	1.41 (1.01-1.96)*

HR for mortality		Body mass index (kg/m^2)					
		15.0-20.0	20.0-22.5	22.5-25.0	25.0-27.5	27.5-30.0	30.0-35.0
Male	Adjusted model	2.57 (1.75-3.75)**	1.46 (1.04-2.05)*	1.20 (0.88-1.64)	1.16 (0.84-1.61)	1 (reference)	1.35(0.87-2.09)
	Adjusted model + WHR	2.59 (1.76-3.82)**	1.52 (1.08-2.15)*	1.23 (0.90-1.67)	1.18 (0.85-1.64)	1 (reference)	1.34 (0.86-2.09)
	Adjusted model + WHR and MIS	2.45 (1.66-3.62)**	1.53 (1.08-2.16)*	1.24 (0.90-1.69)	1.19 (0.86-1.64)	1 (reference)	1.31 (0.84-2.03)
Female	Adjusted model	1.59 (0.99-2.55)	1.75 (1.14-2.69)*	1.42 (0.93-2.17)	1.33 (0.86-2.06)	1.33 (0.83-2.14)	1 (reference)
	Adjusted model + WHR	1.69 (1.04-2.73)*	1.82 (1.18-2.81)*	1.50 (0.98-2.29)	1.34 (0.86-2.09)	1.38 (0.86-2.21)	1 (reference)
	Adjusted model + WHR and MIS	1.65 (1.02-2.68)	1.83 (1.18-2.82)	1.51 (0.99-2.32)	1.37 (0.88-2.13)	1.40 (0.87-2.25)	1 (reference)

Values expressed as hazard ratio (HR) and 95% confidence interval (CI).

Adjusted model: adjusted for age, gender, eGFR, Upcr log, diabetes, cardiovascular disease, smoker, cancer, severe liver disease, hypertension, hemoglobin, cholesterol log, glycosylated hemoglobin, albumin, CRP ln and phosphorus.

*P < 0.05 compared with reference metabolic syndrome component category.

**P < 0.001 compared with reference metabolic syndrome component category.

Abbreviations: HR: hazard ratio, BMI: Body mass index, MIS: malnutrition-inflammation syndrome, WHR: Waist-to-hip ratio, eGFR: estimated glomerular

filtration rate, Upcr: urine protein and creatinine ratio, CRP: C-reactive protein.

Supplement Table S7. Odds ratios for malnutrition-inflammation according to body mass index and waist-to-hip ratio

OR for malnutrition-inflammation		BMI (kg/m^2)					
		15.0-20.0	20.1-22.5	22.6-25.0	25.1-27.5	27.6-30.0	30.1-35.0
Male	Adjusted model	13.0 (6.3-26.8)*	1.32 (0.81-2.15)	0.97 (0.63-1.49)	0.97 (0.63-1.49)	1 (reference)	1.46 (0.81-2.64)
	Adjusted model + WHR	10.9 (5.2-22.9)*	1.21 (0.73-2.00)	0.94 (0.61-1.46)	1.06 (0.68-1.66)	1 (reference)	1.51 (0.83-2.75)
Female	Adjusted model	12.8 (6. 9-23.7)*	1.86 (1.16-2.99)*	1.36 (0.86-2.14)	1 (reference)	1.36 (0.86-2.14)	1.49 (0.81-2.76)
	Adjusted model + WHR	11.8 (6.3-22.1)*	1.75 (1.08-2.82)*	1.33 (0.84-2.11)	1 (reference)	1.30 (0.75-2.26)	1.58 (0.85-2.94)

OR for malnutrition-inflammation		Waist-to-hip ratio				
		Q1	Q2	Q3	Q4	Q5
Male	Adjusted model	2.07 (1.38-3.12)#	1 (reference)	0.96 (0.64-1.43)	0.98 (0.65-1.46)	0.98 (0.65-1.46)
	Adjusted model + BMI	1.73 (1.13-2.64)#	1 (reference)	1.05 (0.70-1.58)	1.06 (0.70-1.60)	1.01 (0.66-1.56)
Female	Adjusted model	2.22 (1.39-3.54)#	1.39 (0.89-2.18)	1 (reference)	1.14 (0.73-1.79)	1.14 (0.73-1.79)
	Adjusted model + BMI	1.75 (1.06-2.87)#	1.38 (0.86-2.20)	1 (reference)	1.16 (0.72-1.86)	1.03 (0.63-1.69)

Values expressed as odds ratio (OR) and 95% confidence interval (CI).

Adjusted model: adjusted for age, gender, eGFR, Upcr log, diabetes, cardiovascular disease, smoker, cancer, severe liver disease, hypertension, hemoglobin, cholesterol log, glycosylated hemoglobin, albumin, CRP ln and phosphorus.

*$P < 0.001$ compared with reference BMI category.

#$P < 0.05$ compared with reference WHR category.

Abbreviations: BMI: Body mass index, HR: hazard ratio, WHR: waist-to-hip ratio, eGFR: estimated glomerular filtration rate, Upcr: urine protein and creatinine ratio, CRP: C-reactive protein.
